# Evolution of termination codons of proteins and the TAG-TGA paradox

**DOI:** 10.1038/s41598-023-41410-z

**Published:** 2023-08-31

**Authors:** Mária Trexler, László Bányai, Krisztina Kerekes, László Patthy

**Affiliations:** Institute of Enzymology, Research Centre for Natural Sciences, Budapest, 1117 Hungary

**Keywords:** Biochemistry, Evolution, Genetics, Molecular biology

## Abstract

In most eukaryotes and prokaryotes TGA is used at a significantly higher frequency than TAG as termination codon of protein-coding genes. Although this phenomenon has been recognized several years ago, there is no generally accepted explanation for the TAG-TGA paradox*.* Our analyses of human mutation data revealed that out of the eighteen sense codons that can give rise to a nonsense codon by single base substitution, the CGA codon is exceptional: it gives rise to the TGA stop codon at an order of magnitude higher rate than the other codons. Here we propose that the TAG-TGA paradox is due to methylation and hypermutabilty of CpG dinucleotides. In harmony with this explanation, we show that the coding genomes of organisms with strong CpG methylation have a significant bias for TGA whereas those from organisms that lack CpG methylation use TGA and TAG termination codons with similar probability.

## Introduction

In the human genome, the fraction of synonymous codons changes monotonically with the GC content of coding sequences: with increasing the GC content the codons that are richer in GC within their synonymous family increase their fraction and, accordingly, the occurrence of those richer in AT decreases. This is true for both sense and stop codons, showing that the stop site (TAA, TAG, TGA) is affected by the same mutational processes as sense codons^1^.

Interestingly, although TGA and TAG have the same base composition, TGA is used at a higher frequency than TAG, and this bias has been claimed to hold for both prokaryotes and eukaryotes^1–4^. Although the strong bias favoring TGA over TAG has been recognized several years ago, there is no clear consensus as to what explains this phenomenon. Recently Ho and Hurst^4^ have suggested that in species with strong G + C-biased gene conversion (gBGC), such as mammals and birds, the high usage and conservation of TGA is best explained by an A + T → G + C repair bias, but this hypothesis fails to explain TGA enrichment in other G + C-rich genomes. The authors have pointed out that resolution of this paradox may provide insights into either an unknown but common selective preference or an unrecognized complexity to the action of G + C-biased gene conversion. The authors considered the resolution of this paradox of major importance, as it reflects “something profound about genome evolution that we do not currently understand”.

Explanations for the TAG-TGA paradox include models that assume that TGA may have significant selective advantage over TAG as termination codon. It is now well established that the three stop codons are not fully synonymous as they differ in efficiency to terminate translation^5^. TAA appears to be the most efficient, whereas TGA is the least efficient with significant readthrough due to misinterpretation of the stop codon. As TAA is the most effective as a termination codon, TAA codons are conserved by purifying selection in all domains of life, particularly in highly expressed genes^5,6^. Although the context of stop codons has a major influence on their readthrough^7–9^, in general, readthrough increases in the order TAA < TAG <  < TGA.

In view of the foregoing, a possible explanation for the preference of TGA over TAG as termination codon is that there is positive selection for TGA since its readthrough provides a means for proteome expansion^10^. This “adaptive hypothesis” assumes that stop codon readthrough is an important, regulated mechanism for generating proteome diversity as it allows the formation of additional, C-terminally modified protein variants. Investigations in the last decade have revealed that translational stop codon readthrough appears to be more prevalent in Metazoa and Fungi than previously recognized^11–13^. Stop codon readthrough events are also widespread in bacteria. Analyses of premature stop codons of prokaryotic protein-coding genes have revealed that nonsense substitutions are relatively common since—thanks to readthrough—they do not necessarily cause pseudogenization^14^. The benefits and the possible evolutionary significance of functional readthrough of natural termination codons are illustrated by some studies on bacterial populations. It has been shown that heterogeneity of readthrough among single cells may provide advantages to the microbial population by enhancing phenotypic diversity and facilitating adaptation to the changing environment and that single cells with high readthrough levels are more adapted to tolerate stress conditions^15,16^. The functional significance of readthrough is most evident in cases where programmed stop codon readthrough is used to generate peroxisomal isoforms of cytosolic enzymes. For example, it has been shown that readthrough of the leaky UGA codon of NAD-dependent lactate dehydrogenase B and NAD-dependent malate dehydrogenase 1 results in C-terminally extended protein variants containing a peroxisomal targeting signal^7,17,18^.

It should be mentioned, however, that a competing hypothesis assumes that stop-codon read-through arises mostly from molecular errors and is largely nonadaptive^19^. As arguments in favor of their error hypothesis and against the adaptive hypothesis the authors point out that read-through rates decrease with gene expression levels, read-through motifs are avoided in highly expressed genes and that read-through regions do not show increased sequence conservation. There are several examples illustrating the deleterious nature of C-terminal extensions resulting from readthrough, questioning the general validity of the “adaptive hypothesis” as an explanation for the codon usage bias of wild type stop codons. It has been shown that some 3’UTR-encoded readthrough peptides mark their resulting products for destruction, mitigating their deleterious effects^20^. It has also been shown that some stop codon read-through mutant proteins are degraded via the ubiquitin–proteasome system^21^.

It must be emphasized, however, that the adaptive and error hypotheses for stop codon readthrough are not necessarily mutually exclusive. Assuming that—on a proteome scale—the selective benefits of readthrough may be more significant than its potential deleterious effects, readthrough could still provide an explanation for the TAG-TGA paradox.

In the present work we have tested the validity of the adaptive hypothesis of the TAG-TGA paradox of human genes by analyzing nonsense mutations accumulated during cancer evolution in different groups of cancer genes that are expected to be selected for or against stop codon readthrough. The rationale of our approach was that in the case of tumor suppressor genes readthrough of inactivating stop codons would counteract the effect of the driver mutation, thus in this case selection would favor TAA and TAG over the leaky TGA codon. Conversely, in the case of oncogenes and tumor essential genes readthrough of stop codons may rescue these pro-oncogenic genes from inactivation by nonsense mutations, therefore selection is expected to favor the leaky TGA over the more efficient TAA and TAG termination codons.

Analysis of mutation data of cancer tissues has revealed that the relative rates of the three nonsense mutations of tumor suppressor genes did not differ significantly from those of oncogenes and tumor essential genes or passenger genes. These observations suggest that differences in termination efficiency are unlikely to explain the TAG-TGA paradox.

Unexpectedly, in the case of all gene groups, the rates of TGA mutations were significantly higher than those expected based on codon frequencies of the genes and the known mutation bias of single base substitutions of tumors and, as a consequence, the fraction of TGA was higher than that of TAG. To get an insight into the source of this excess of TGA, we have surveyed the mutation data of the genes. These analyses revealed that out of the eighteen sense codons that can give rise to a nonsense codon by single base substitution, the CGA codon was exceptional, in as much as it gave rise to a stop codon (the TGA codon) at a significantly higher rate than the other codons, suggesting that the inherent hypermutability of the CpG dinucleotide of CGA codons may underly the TAG-TGA paradox.

An implicit prediction of this hypothesis is that organisms with non-methylated genomes are not expected to favor TGA over TAG as termination codons. In harmony with this prediction, we show that the TAG-TGA distinction parallels the the evolutionary gain and loss of CpG methylation.

## Results and discussion

### Codon usage bias and mutation bias of single base substitutions do not explain why TGA is more abundant than TAG as termination codon of human genes

Eighteen of the 61 sense codons can give rise to stop codons through single base substitutions. The different amino acids and their stopogenic sense codons differ markedly in the type of stop codon they can generate by single base substitution (Supplementary Table 1). For example, codons of tyrosine (TAC, TAT) can give rise to both TAA and TAG, the tryptophan codon (TGG) can generate both TGA and TAG, whereas substitutions of codons of cysteine (TGC, TGT) and arginine (AGA, CGA) can lead only to the formation of TGA (Supplementary Table 1). Accordingly, the amino acid composition and codon usage of proteins have a marked influence on the relative probability of the formation of the three stop codons. Taking into account the frequency of stopogenic codons in the human proteome (assuming that there was no difference in the probability of the substitution classes), the fractions of termination codons are expected to be fTAA = 0.3073; fTAG = 0.4448; fTGA = 0.2478.

Thus, based on just the frequency of stopogenic codons in the human proteome, single base substitutions leading to TAG would far exceed those leading to TGA, just the opposite of what we find in the case of natural termination codons of the human proteome. As shown by Ho and Hurst^4^, in the case of the human proteome, fTAA = 0.2692; fTAG = 0.2096; fTGA = 0.5212.

Differences in the probability of the six substitution classes have a major impact on the relative probability of the three nonsense mutations (Supplementary Table 1). It is noteworthy, however, that although mutation bias may affect the relative proportion of TAA vs. TGA and TAG, the relative probability of the TAG –TGA mutations are insensitive to such differences, as they have the same composition.

In summary, the amino acid composition, codon usage bias or mutation bias do not provide an explanation as to why TGA is much more abundant than TAG as termination codons of human protein-coding genes.

### Nonsense mutation spectra of tumor suppressor genes, oncogenes, tumor essential genes and passenger genes are similar, suggesting that differences in termination efficiency have no major impact on the choice of nonsense mutations

To test whether the differences in the efficiency of TAG and TGA as signals for translation termination play a role in the differences in the abundance of TAG and TGA, we have analyzed nonsense mutations accumulated during cancer evolution in cancer genes that are expected to be most sensitive to truncation by nonsense mutations. In the case of tumor suppressor genes (positively selected for nonsense mutations) readthrough of inactivating stop codons would dampen the effect of the driver mutation, therefore selection should favor TAG over the leaky TGA codon. Conversely, in the case of oncogenes and tumor essential genes that are known to be negatively selected against nonsense mutations^22^, readthrough of stop codons may rescue these pro-oncogenic genes from inactivation therefore selection should favor the leaky TGA codon over TAG. In these studies we have used the lists of oncogenes (OGs) and tumor suppressor genes (TSGs) defined by Vogelstein et al.^23^. The list of tumor essential genes (TEGs) consisted of genes identified by Bányai et al.^22^. As a control group, we have used the list of selectively neutral passenger genes (PGs) characterized by Bányai et al. and Bányai et al.^22,24^.

We have retrieved the mutation data of these genes from the COSMIC database and identified all single base substitutions resulting in nonsense mutations. The primary data of nonsense mutations for the four sets of proteins are found in Supplementary file 1. This dataset contains information on the positions and identities of the wild type amino acids and wild type codons, the single base substitutions, the resulting stop codon and the number of times the given nonsense mutation has been observed. The observed spectra of the three types of nonsense mutations were determined by including the recurrence number of the given mutation in the calculations (Supplementary file 2).

Since amino acid composition, codon frequency and mutation bias has a major impact on the spectrum of nonsense mutations (see section "Codon usage bias and mutation bias of single base substitutions do not explain why TGA is more abundant than TAG as termination codon of human genes"), for each protein-coding gene we have calculated the fTAA, fTAG and fTGA values expected on the basis of their stopogenic codon frequency and the mutation bias characteristic of tumor cells (Supplementary file 3).

**Figure 1:**
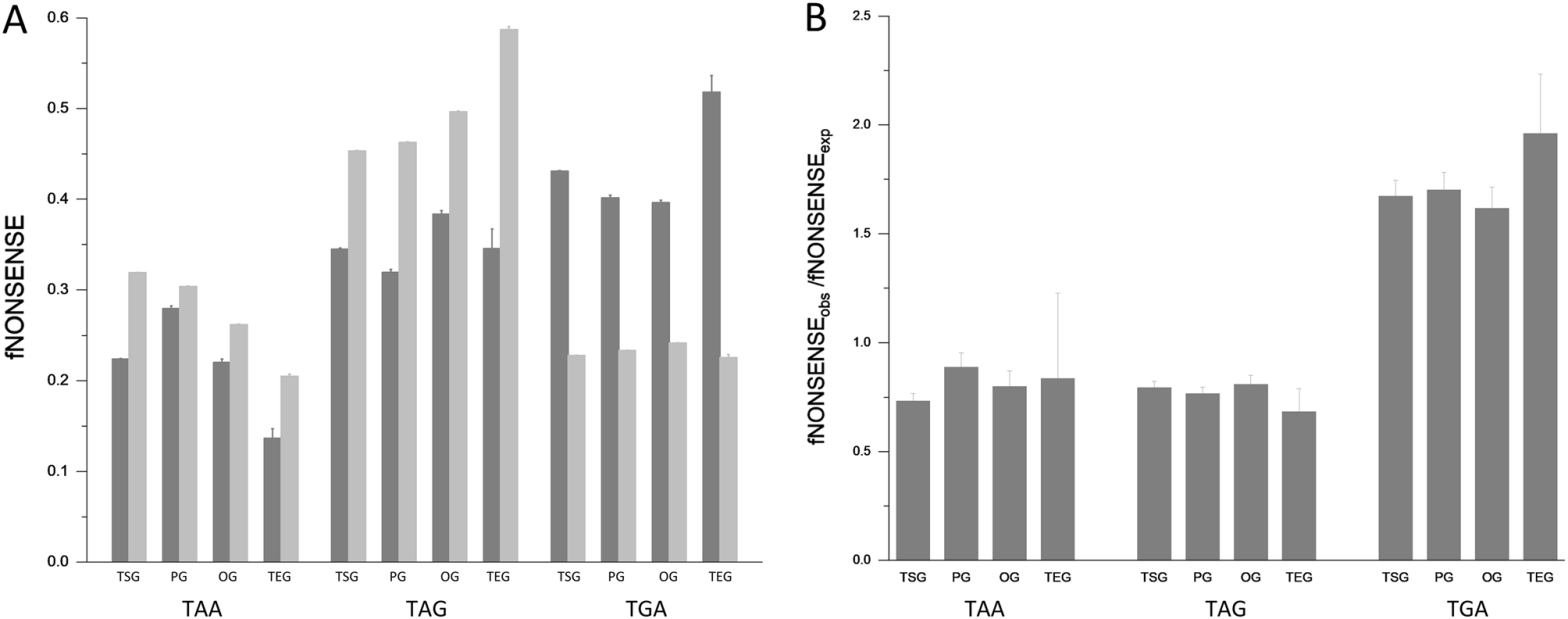
Comparison of the observed and expected spectra of nonsense substitutions on tumor suppressor genes (TSG), passenger genes (PG), oncogenes (OG) and tumor essential genes (TEG). (**A**) The light grey columns represent the expected fTAA, fTAG and fTGA values, the dark grey columns show the values observed in cancer tissues. (**B**) The figure shows the ratios of the observed and expected fTAA, fTAG and fTGA values for the four groups of genes*.*

Comparison of the ratios of observed and expected fTAA, fTAG and fTGA values revealed no significant differences among tumor suppressor genes, oncogenes and tumor essential genes (Fig. 1, Supplementary file 4), suggesting that the differences in termination efficiency of the three stop codons has no major impact on the pattern of nonsense mutations observed in cancer tissues.

### The observed rates of single base substitutions leading to TGA are significantly higher than those expected based on codon usage and mutation bias

**Figure 2:**
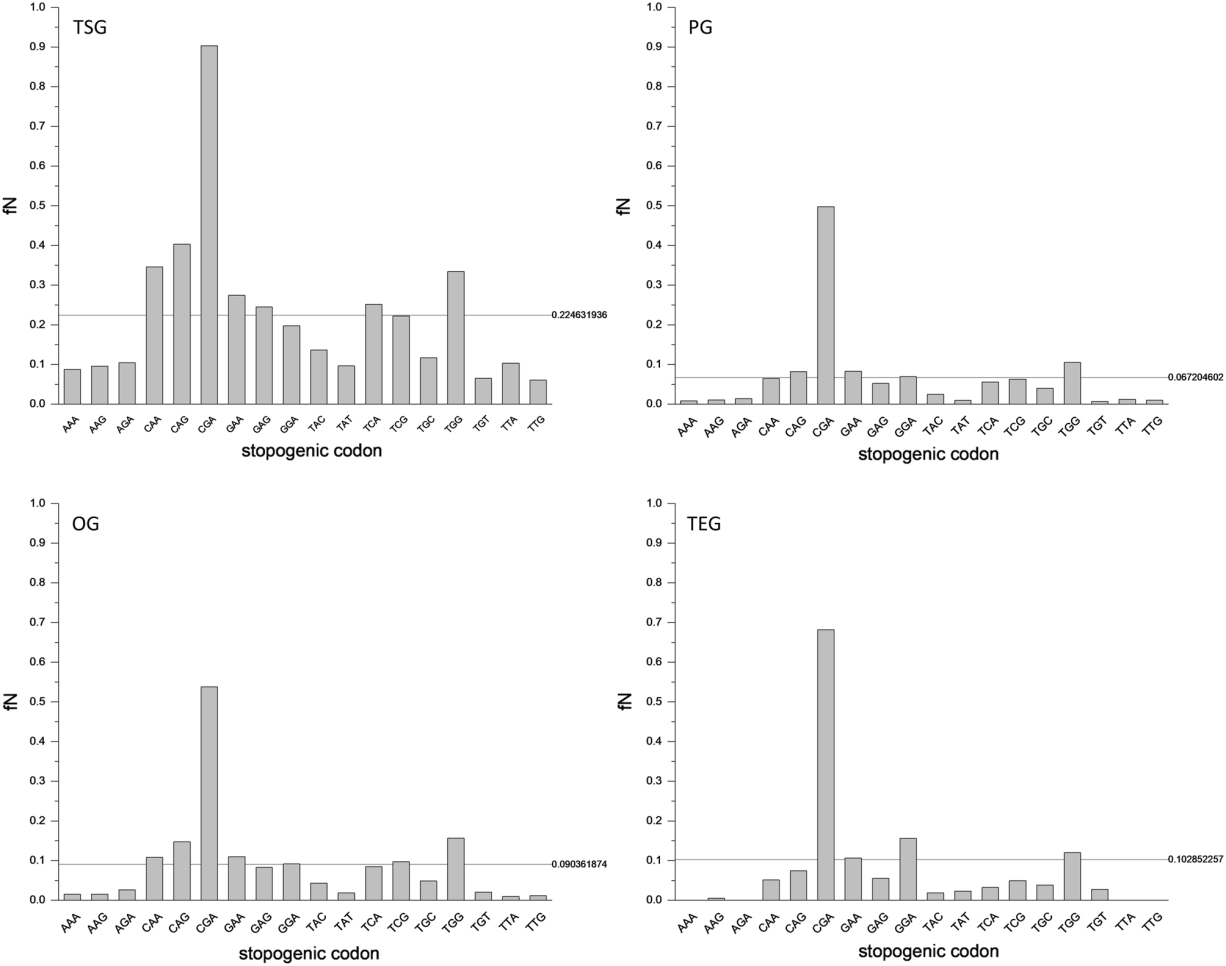
Frequencies of nonsense mutations of different stopogenic sense codons in tumor suppressor genes (TSG), passenger genes (PG), oncogenes (OG) and tumor essential genes (TEG) in cancer. The ordinates show fN representing the fraction of stopogenic codons that experienced a nonsense mutation at least once, corrected for codons that can give rise to two nonsense codons (e.g. TAC, TGG). The abscissa lists the stopogenic codons. In each panel the horizontal lines indicate the average values for the eighteen stopogenic codons*.*

**Figure 3:**
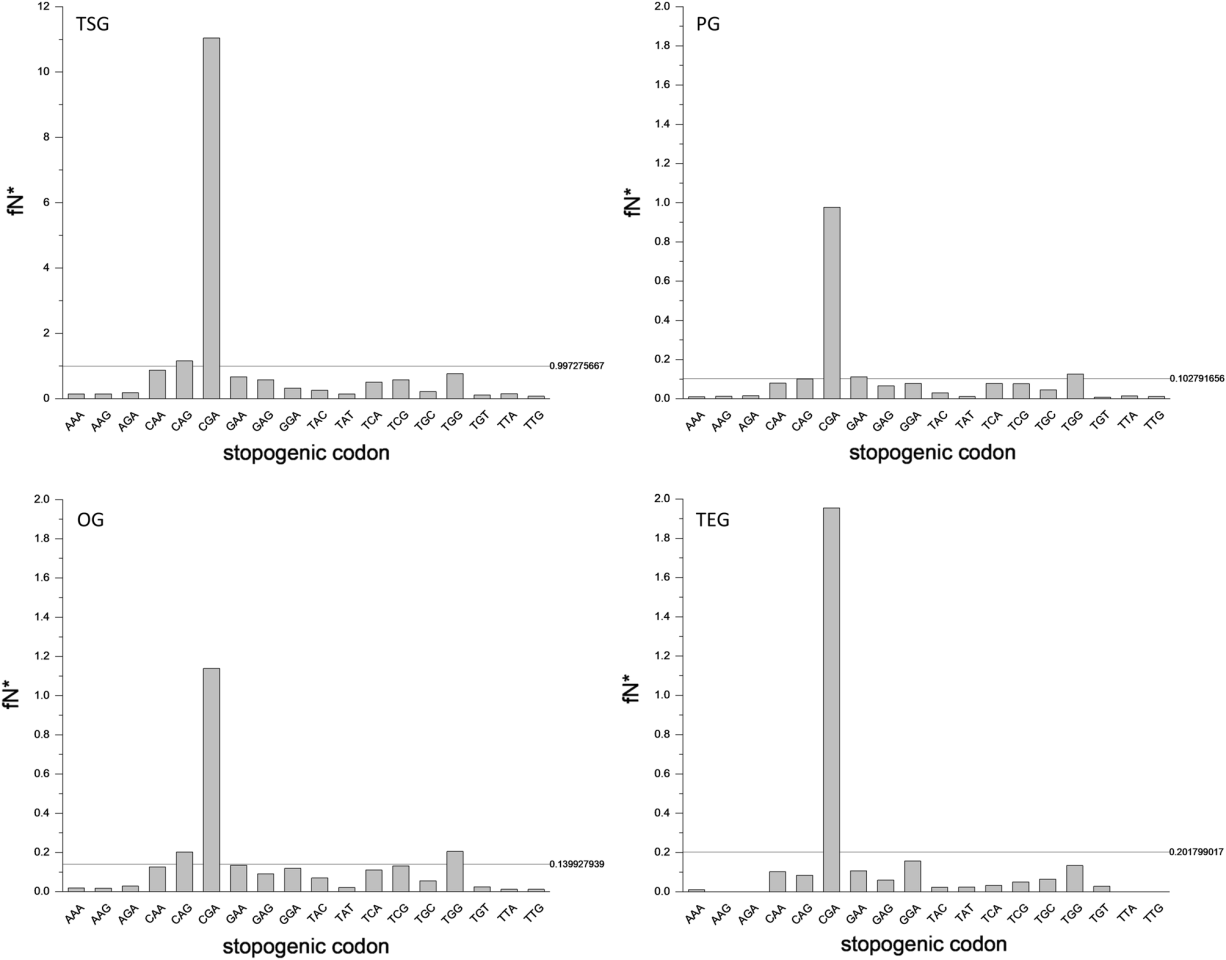
Frequency and recurrence of nonsense mutations of different stopogenic sense codons in tumor suppressor genes (TSG), passenger genes (PG), oncogenes (OG) and tumor essential genes (TEG) in cancer. The ordinates show fN*, the fraction of stopogenic codons that experienced a nonsense mutation at least once (corrected for codons that can give rise to two nonsense codons), multiplied by the number of times the mutation was observed. The abscissa lists the stopogenic codons. In each panel the horizontal lines indicate the average values for the eighteen stopogenic codons.

Comparison of the observed and expected spectra of nonsense substitutions in cancer tissues revealed that the observed fTGA values are 1.689-fold higher than those expected based on codon usage and mutation bias of single base substitutions in cancer (Supplementary file 4, Fig. 1). To get an insight into the possible source of this excess of TGA, we have surveyed the spectrum of nonsense mutations observed in cancer (Supplementary file 1). Our analyses have revealed that the eighteen stopogenic sense codons differed markedly in their probability to give rise to a stop codon (Fig. 2). In all four groups of genes the CGA codon gave rise to a stop codon (TGA) at a significantly higher rate than the other codons (Supplementary file 5). The exceptionally high rate of the CGA to TGA mutation is even more evident when the recurrence of nonsense mutations is also taken into account (Fig. 3, Supplementary file 6).

Our studies on nonsense mutations in the human germline (Supplementary file 7) have shown that the relative probability of the use of stopogenic codons is very similar to that observed in the case of somatic mutations in cancer, indicating that germline and somatic cells share the hypermutability of CGA codons. In all four groups of genes (TSGs, OGs, TEGs and PGs) the CGA codon gave rise to a stop codon (TGA) at a significantly higher rate than the other stopogenic codons (Supplementary file 8, Supplementary Fig. 1).

### Hypermutability of CpG dinucleotide of the CGA codon is responsible for the excess of CGA → TGA mutations

It should be noted that the CGA to TGA mutation involves the mutation of a cytosine of a CpG dinucleotide that is known to have inherent hypermutability in methylated genomes. In mammalian genomes CpG dinucleotides are methylated at position 5 of cytosine and spontaneous deamination of methylated cytosine yields thymine, thus leading to the dinucleotide TpG. Thanks to spontaneous deamination, CpG dinucleotides of human genes are known to undergo germ-line transition to TpG at frequencies six to seven times the base mutation rate^25^.

Since CGA is the only stopogenic codon that gives rise to a stop codon through mutation of a CpG dinucleotide, we suggest that the methylation-based hypermutability of CGA codons and the increased rate of TGA formation may provide a clue for understanding the TAG-TGA paradox.

Nevertheless, the fTGA/fTAG ratios of nonsense mutations observed in cancer (fTGA/fTAG ~ 1.236, Supplementary file 2, Fig. 1,) are lower than the values for natural termination codons of human protein-coding genes (fTGA/fTAG ~ 2.49) calculated from the data of Ho and Hurst^4^ (Table 1). It should be pointed out, however, that there is a major difference between somatic nonsense substitutions arising de novo in cancer tissues and natural termination codons of protein-coding genes: the former appear during the lifespan of the organism, whereas termination codons are the results of the long evolutionary history of protein-coding genes.

**Table 1:** Nonsense codon usage bias in different datasets of protein-coding genes.

Dataset	Proteins	fTAA	fTAG	fTGA	TGA/TAG	Reference of dataset
Human proteome	19,850	0.269	0.209	0.521	2.487	Ho and Hurst^4^
Human somatic nonsense mutations	220	0.228	0.345	0.427	1.236	Present work
Human out of frame stop codons	220	0.238	0.165	0.598	3.631	Present work
De novo human proteins	24	0.167	0.250	0.583	2.333	Xie et al.^37^
De novo human proteins	73	0.203	0.230	0.568	2.471	An et al*.*^38^
De novo human proteins	97	0.351	0.154	0.494	3.214	Sandmann et al.^39^
Mouse proteome	22,515	0.287	0.225	0.487	2.165	Subramanian et al.^57^
De novo mouse proteins	26	0.307	0.192	0.500	2.600	Ruiz-Orera *et.al*.^36^
Fly proteome	13,930	0.406	0.344	0.249	0.724	Subramanian et al.^57^
De novo fly proteins	34	0.441	0.323	0.235	0.728	Heames et al.^44^
De novo fly proteins	20	0.450	0.250	0.300	0.833	Begun et al.^45^
Worm proteome	21,095	0.463	0.167	0.369	2.210	Subramanian et al.^57^
De novo worm proteins	46	0.500	0.152	0.347	2.285	Lee et al.^41^
Yeast proteome	5989	0.473	0.230	0.296	1.288	Subramanian et al.^57^
De novo yeast proteins	169	0.426	0.254	0.319	1.255	Blevins et al.^40^

### Evolutionary origin of termination codons

According to the currently accepted evolutionary models, protein-coding genes evolve from proto-genes that emerge de novo when non-genic sequences become transcribed and translated^26,27^. Phylostratigraphy studies suggest that de novo evolution of genes has occurred continuously throughout evolutionary time and should therefore be considered as a general mechanism for the emergence of new gene functions^28^.

**Figure 4:**
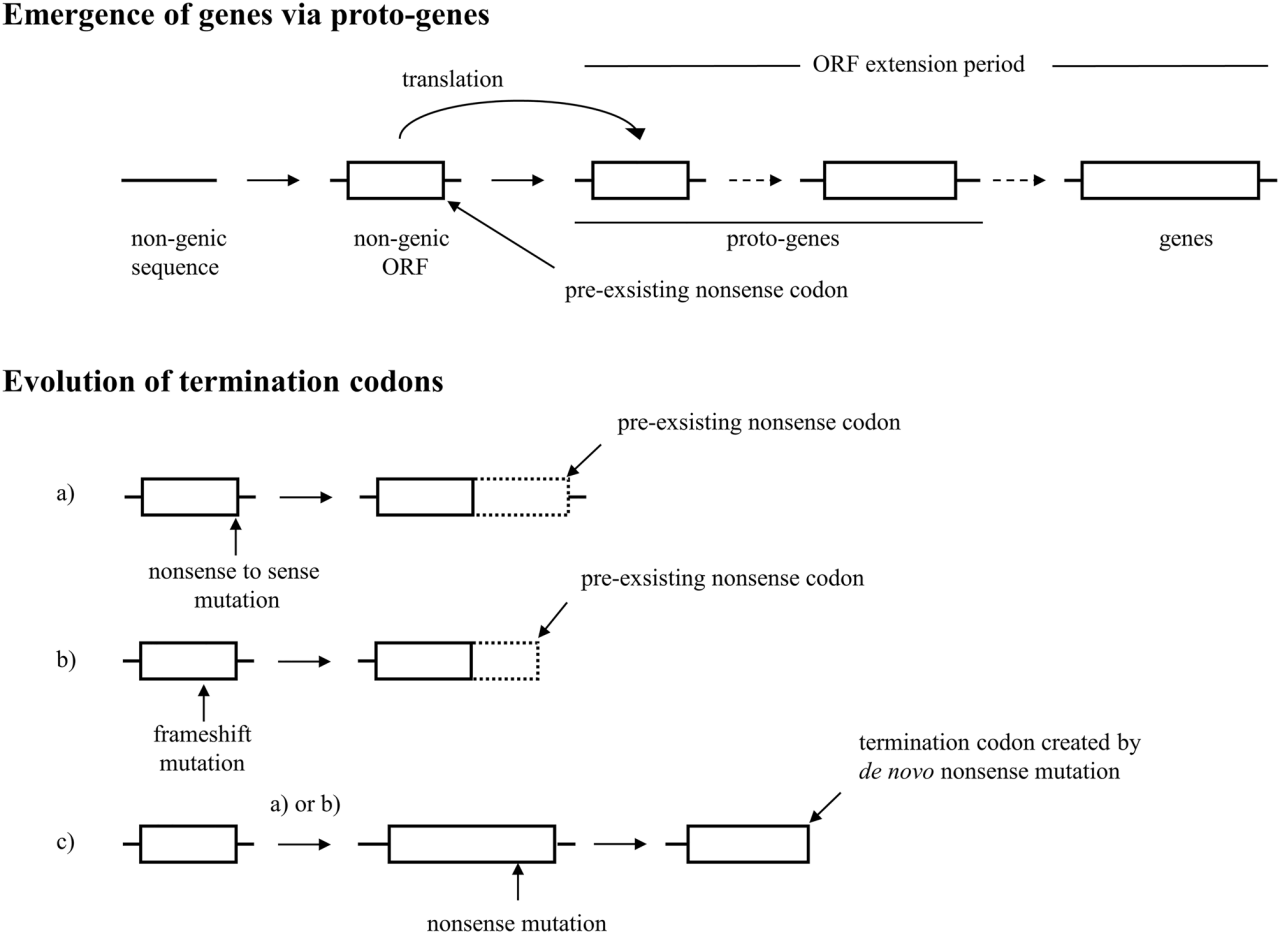
Evolutionary origin of the termination codons of protein-coding genes. The upper part of the figure shows a modified version of the model proposed by Carvunis et al.^26^ for the gradual emergence of protein-coding genes in non-genic sequences via proto-genes. A key aspect of the model is that the short ORFs of proto-genes utilize pre-existing termination codons, increase in length over evolutionary time and this process also involves changes in the position of termination codons. The lower part of the figure shows different scenarios for changes in the termination codons of ORFs during the evolution of genes. (**a**) The termination codon is lost through nonsense to sense mutations and pre-existing hidden nonsense codon downstream of the original ORF serves as the new termination codons of the C-terminally extended ORFs. (**b**) The original termination codon is lost as a result of mutations causing frame-shift (indels, changes in splicing) and pre-existing nonsense codons (out of frame of the original ORF) serve as the new termination codons of the mutant ORFs. (**c**) Sense to nonsense mutations upstream of the actual termination codon creates the new termination codons of the mutant ORFs.

The proto-genes that emerge de novo utilize randomly occurring, pre-existing start and stop codons, therefore they are likely to encode very short polypeptides, but during proto-gene to gene transition the open reading frames lengthen significantly^26,29^. As part of the latter process the original termination codons of the nascent proto-genes may be replaced by new ones in a complex interplay of nonsense to sense, sense to nonsense substitutions and frame-shift mutations affecting the C-terminal regions (Fig. 4). Genes may recruit pre-existing hidden stop codons as termination codons through mutations that cause shifts of reading frame (indels, changes in splicing) or nonsense to sense substitutions (Fig. 4a,b), but new termination codons may also be gained through de novo nonsense mutations affecting the translated region (Fig. 4c).

Our analysis of the hidden stop codons of human protein-coding genes has revealed that the TGA codon is the most abundant* (*Supplementary file 9, Table 1). Interestingly, the TGA/TAG ratio of out-of-frame stop codons (3.631) is significantly higher than in the case of nonsense mutations (~ 1.236) and even exceeds the ratio of wild type, in-frame termination codons (2.487, Table 1).

It must be emphasized that the preponderance of hidden, pre-existing TGA codons and the increased rate of CGA → TGA nonsense mutations have common roots: hypermutability of CpG. It has long been known that—thanks to its hypermutability—the CpG dinucleotide is strongly underrepresented in methylated genomes and that the CpG deficit correlates with increased abundance of TpG dinucleotides^30–32^. In fact, CpG hypermutability plays a major, general role in determining the amino acid composition and codon usage of proteins. It has been shown that there is a universal trend of amino acid gain–loss that is caused by CpG hypermutability. CpG hypermutability increases the frequency of amino acids coded by codons with TpG dinucleotides but decreases the frequency of amino acids coded by codons with CpG dinucleotides^33^. CpG hypermutability is also one of the determining factors of codon preferences among synonymous codons, in as much as in the case of codon families synonymous codons containing CpG tend to be the rarest, whereas those containing TpG are the most frequent^34,35^.

The question, however, remains: why is the TAG-TGA difference more pronounced in the case of pre-existing hidden stop codons and wild type termination codons of human protein-coding genes than in the case of de novo somatic nonsense mutations that arose during the evolution of cancer (Table 1). The most plausible explanation is that it stems from the difference in the time-scale of the events. As mentioned above (Section "Hypermutability of CpG dinucleotide of the CGA codon is responsible for the excess of CGA→TGA mutations"), there is a major difference between somatic nonsense substitutions arising de novo in cancer tissues and hidden and functional termination codons: the latter are the results of the long evolutionary history of the organisms, whereas the former are recent, they appear during their lifespan. The higher levels of TpG dinucleotides and hidden TGA codons in genomes accumulated as a result of long evolutionary periods of CpG hypermutability that have also depleted CpG dinucleotides, whereas de novo somatic mutations affect genomes in which CpG dinucleotides are significantly underrepresented.

### TAG-TGA usage of nascent protein-coding genes

There is an increasing body of evidence that de novo gene origination is an important mechanism for the formation of new genes and that recently evolved de novo genes may provide an explanation for some species- or lineage-specific trait^28^. As discussed above, proto-genes may recruit pre-existing hidden stop codons as termination codons, but during the proto-gene to gene transition new termination codons may be gained both through de novo nonsense mutations and through acquisition of hidden stop codons (Fig. 4). However, the relative contribution of pre-existing and de novo nonsense codons to termination codons of protein-coding genes is unclear.

To get an insight into the origin of termination codons of new, nascent protein-coding genes we have analyzed the stop codon usage of de novo genes defined in recent studies. Ruiz-Orera et al. have identified several translated de novo protein-coding genes unique to mouse^36^; in similar studies on the human genome, Xie et al., An et al. and Sandmann et al. have identified several hominoid-specific de novo protein-coding genes^37–39^. In many cases the transcripts of these genes were originally annotated as long non-coding RNAs (lncRNAs) because they lacked conserved open reading frames. The encoded proteins are usually much shorter than average and show no selective constraints, suggesting that their features are determined primarily by the chance occurrence of pre-existing start and stop codons in lncRNAs^29^. For example, the median protein size of the de novo genes identified by Ruiz-Orera et al. was 44 *versus* the 412 amino acids of conserved ORFs^36^.

Our analysis of the transcripts of de novo murine and human genes with proteomic evidence for the translation of the ORFs has shown that these young “proto-genes” display pronounced preference of TGA over TAG (TGA/TAG > 2, Supplementary file 10, Table 1). In the case of de novo human protein-coding genes, the TGA/TAG ratio (2.4–3.2) was higher than the value for nonsense mutations (1.24), but lower than the value for hidden stop codons (3.63), suggesting that both pre-existing and de novo nonsense codons contributed to their termination codons. It is noteworthy that the TGA/TAG ratio (3.21) of the youngest de novo microproteins translated from short open reading frames^39^ is quite close to the value for hidden stop codons (3.63), suggesting that these microproteins use primarily pre-existing stop codons as termination codons. Despite these age-dependent minor differences, the TGA/TAG ratios of young and old human protein-coding genes are remarkably similar.

The similarity of termination codon usage bias of young and old proteins also holds for eukaryotes with less significant CpG methylation and CpG hypermutability (see section "CpG hypermutability and the TAG-TGA paradox of termination codons"). Our analyses of de novo genes of *Saccharomyces cerevisae*, *Caenorhabditis elegans* and *Drosophila melanogaster*^40–45^ has revealed that the termination codon usage of de novo genes is similar to that of their proteome (Supplementary file 10; Table 1).

### CpG hypermutability and the TAG-TGA paradox of termination codons

The CpG hypermutability-based explanation of the TAG-TGA paradox predicts that the presence or absence of CpG hypermethylation should correlate with the presence or absence of the TAG-TGA distinction in different groups of organisms.

Although information on hypermethylation and hypermutability of CpG in prokaryotes is much more limited than for Eukaryotes, there is evidence for its role in genome evolution of Bacteria and Archaea. It has been shown that CpG-specific DNA methyltrasnsferases do exist in Eubacteria and Archaea and that CpG dinucleotides are significantly underrepresented and TpG dinucleotides overrepresented in some bacteria as expected based on CpG hypermutability^33,46^ Analyses of hidden (out-of-frame) codons of bacterial genomes revealed a general strong bias toward TGA but against TAG, with a systematic excess of TGA codons in the organisms studied^47,48^, suggesting that hypermutability of CpG dinucleotide might contribute to termination codon usage bias in prokaryotes*.*

CpG hypermethylation is widespread in Eukaryotes, nevertheless there is evidence for group-specific differences and that CpG hypermethylation and thus CpG hypermutability has been lost in several lineages^49,50^. Although DNA methyltransferases (DNMTs) and DNA methylation are present in all groups of Ecdysozoa, some groups have apparently lost these enzymes and DNA methylation^51^. Genome-wide and coding levels of DNA methylation are relatively high in Lepidoptera, Coleoptera, Hymenoptera and Hemiptera, with the highest levels observed in Blattodea, but Diptera (including *Drosophila melanogaster*) have lost CpG methylation^52^. Similarly, members of the DNMT family and DNA methylation are present in Nematoda, but the five Clades of these worms have experienced differential loss of DNA methyltransferases^51^ Clade I, including *Trichinella spiralis*, has retained DNMTs and there is evidence for DNA methylation in this parasitic nematode^53^. In the case of Clade V of nematodes that contains *Caenorhabditis elegans*, DNMT2 could be detected in 5 species but most species had no DNMTs^51^. The absence of DNMTs in *C. elegans* is in harmony with ealier observations that this model organism shows zero DNA methylation^54^. Although DNA methylation is present in Fungi, such as *Neurospora crassa*, it has been lost in *Saccharomyces cerevisiae, Schizosaccharomyces pombe*, and *Aspergillus nidulans*^50^. Yeast species do not methylate their DNA and a distinctive CpG transition rate is absent in yeast^55,56^.

Thus, in view of our hypothesis that the TAG-TGA paradox can be best explained by CpG hypermutability, it is of major interest to see how evolutionary gain and loss, presence or absence of CpG hypermethylation had an impact on the stop codon usage of proteomes. To answer these questions, we have examined the termination codon usage of representative groups of organisms using the Codon Statistics Database of Subramanian et al.^57^.

**Figure 5:**
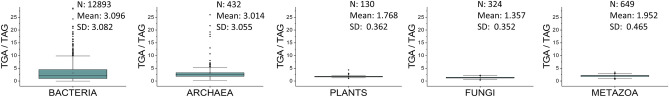
Variation of the TGA/TAG ratios of proteomes of species from different groups of organisms. A. Boxplots of TGA/TAG ratios of Bacteria, Archaea, Plants, Fungi and Metazoa. N: number of species in the taxon; Mean: mean of TGA/TAG values of the species in the taxon; SD: standard deviation of TGA/TAG values.

Analysis of the TGA/TAG ratios of species representing major groups of Eukarya, Archaea and Bacteria (Supplementary file 11) has revealed major differences in codon usage bias of the TGA and TAG termination codons (Table 2, Fig. 5). Whereas in the case of Eukaryotes the SD values of TGA/TAG ratios were relatively low, in the case of Archaea and Bacteria there was extreme variation in TGA/TAG ratios (Supplementary file 11, Table 2 Fig. 5)*.*

**Table 2:** TGA/TAG ratios of different groups of organisms.

Taxon			Species	Mean TGA/TAG	SD
*Bacteria*			12,758	3.127	3.081
	*Deinococcus*		55	6.329	3.556
	*Actinobacteria*		2895	4.748	3.267
	*Proteobacteria*		5351	3.786	3.218
	*Thermotogae*		43	3.576	2.423
	*Firmicutes*		2552	1.399	1.554
	*Cyanobacteria*		163	1.076	1.144
	*Bacteroidetes*		1521	0.899	0.610
	*Mycoplasmatales*		105	0	0
	*Mycoplasmoidales*		2	0	0
	*Spiroplasmataceae*		28	0	0
*Archaea*			432	3.014	3.055
	*Halobacteria*		214	2.617	0.546
	*Methanobacteria*		30	1.217	0.643
	*Methanosarcinales*		32	9.431	8.016
	*Thermococci*		33	3.886	1.116
*Viridiplantae*			127	1.777	0.361
*Fungi*			324	1.357	0.352
			*S. pombe*	0.952	–
			*S. cerevisiae*	1.290	–
*Metazoa*			649	1.952	0.465
	*Porifera*		1	1.645	–
	*Cnidaria*		10	1.579	0.182
	*Mollusca*		10	2.015	0.397
	*Arthropoda*		191	1.452	0.475
		*Diptera*	64	0.974	0.236
			*D. melanogaster*	0.728	–
		* Coleoptera*	11	1.331	0.334
		* Lepidoptera*	23	1.343	0.126
		* Hemiptera*	12	1.753	0.119
		* Hymenoptera*	55	1.811	0.150
		*Blattodea*	2	2.105	0.034
	*Nematoda*		8	2.014	0.625
			*S. ratti*	0.892	–
			*C. elegans*	2.220	–
			*T. spiralis*	3.044	–
	*Platyhelminthes*		4	1.680	0.293
	*Echinodermata*		6	1.670	0.133
	*Chordata*		415	2.198	0.198
		* Branchiostoma*	2	1.289	0.016
		*Tunicata*	*2*	2.166	0.292
		* Vertebrata*	411	2.203	0.188

The TGA/TAG ratios of Bacteria also show marked group-specific differences. In some bacterial taxons (e.g. *Deinococcus, Actinobacteria, Proteobacteria* and *Thermotogae*) there was a strong bias for TGA over TAG, whereas in several other taxons (e.g. *Bacteroidetes, Cyanobacteria and Firmicutes*) there was weak or no preference of TGA over TAG (Supplementary file 11, Table 2, Supplementary Fig. 2). It is noteworthy that *Deinococcus radiodurans,* with an exceptionally high TGA/TAG ratio, possesses a C-5 cytosine DNA methylase that shows eukaryotic type sequence specificity, preferentially methylating CpG and CpC sequences^58^ ; this observation raises the possibility that hypermutability of CpG may have played a role in the high TGA/TAG ratio of *Deinococcus*. However, significant variation of termination codon usage of Bacteria may stem from the fact that stop codons are frequently interpreted as sense codons. For example, in the case of *Mollicutes* (e.g. *Mycoplasma, Spiroplasma*) the TGA codon is read as tryptophan^59,60^ and recent studies have shown that in some uncultured bacteria the TGA codon serves as a fifth glycine codon^61,62^.Recently, Belin and Puigbo^5^ have pointed out that translational selection has a major impact on stop codon usage of bacteria. These authors have analyzed the frequencies of stop codons in a group of highly expressed genes from 196 prokaryotes under strong translational selection and have shown that the occurrence of the three translation termination codons is highly biased, with TAA being the most prevalent in almost all bacteria. The authors have proposed that readthrough efficiency and context effects explain the prevalence of TAA over TAG and TGA, particularly in highly expressed genes.

Archaea also show significant group-specific differences of TGA/TAG ratios. In some taxons of Archaea (e.g. *Methanobacteria*) there is only a weak preference of TGA over TAG, whereas in other groups (e.g. *Halobacteria, Thermococci*) TGA preference is more pronounced (Supplementary file 11, Table 2). *Methanosarcinales* are exceptional in that species in this group have extremely high TGA/TAG ratios (Supplementary file 11, Table 2, Supplementary Fig. 2). Interestingly, *Methanosarcinales* are unique among Archaea as they possess a Pyl-coding system that ensures the insertion of pyrrolysine at TAG codons^63,64^, suggesting that the emergence of the Pyl-coding system had a strong negative influence on the use of TAG as termination codon.

 It seems thus very likely that the use of TGA and TAG as termination codons in prokaryotes is influenced primarily by factors distinct from those operating in Eukaryotes. Whereas in Eukarya the TGA *versus* TAG bias results from CpG methylation and hypermutability, in the case of prokaryotes changes in the function of stop codons may have a more significant impact on their use as termination codons.

The TGA/TAG ratios of major groups of Eukaryotes vary in a narrower range than in prokaryotes. The lowest values of TGA/TAG ratios were observed in the case of Fungi, whereas the highest values are characteristic of Vertebrates (Supplementary file 11, Table 2). This observation is in harmony with the role of CpG hypermutability in the TAG-TAG paradox: hypermutability and depletion of CpG dinucleotides is more pronounced in vertebrates than plants, fungi or invertebrates^65^.

The mean TGA/TAG ratio for non-chordate Metazoa (Porifera, Cnidaria, Nematoda, Arthropoda, Mollusca and Echinodermata) is significantly lower than those of vertebrates (Supplementary file 11; Table 2), consistent with an overall increase in CpG methylation in the Chordate lineage. In the case of Chordates, the average value of TGA/TAG was 2.2 (Table 2). Interestingly, the invertebrate chordate *Branchiostoma* had significantly lower values of TGA/TAG (1.3) than vertebrates. It is worthy of note, that although surveys of the amphioxus genome revealed the presence of the DNA methylation machinery, DNA methyltransferases and methyl-CpG-binding domain proteins, analysis of CpG methylation of the amphioxus genome suggests a bimodal distribution of DNA methylation^66^. Domains of methylated DNA are interspersed with domains of unmethylated DNA, a situation radically different from the globally methylated vertebrate genomes. The mosaic pattern of DNA methylation in amphioxus represents the ancestral condition of the chordate genome, while the global pattern of DNA methylation might be a vertebrate innovation. These observations underline a major difference between genomes of invertebrates and vertebrates. Whereas invertebrate genomes tend to be sparsely methylated, and DNA methylation is mostly targeted to a subset of transcription units (gene bodies), vertebrate genomes are generally globally and heavily methylated. The genomes of Cephalochordates and Tunicates represent different stages in the evolutionary transition of promoter and gene body DNA methylation across invertebrate-vertebrate boundary^67^. Significantly, this transition is also paralleled by an increase in the TGA/TAG ratios of the termination codons of the protein-coding genes of Chordates. There were, however, significant variations of CpG methylation within the various taxonomic groups that were also reflected by their TGA/TAG values. For example, in the case of Fungi, *Schizosaccharomyces pombe* that does not methylate its DNA has no bias for TGA (Supplementary file 11, Table 2). Similarly, in the group of Nematoda, *Strongyloides ratti* that has lost DNMTs had the lowest TGA/TAG value (0.89), whereas *Trichinella spiralis* that methylates its DNA had the highest TGA/TAG values (3.01). Significant variation of TGA/TAG values is also observed in Arthropoda consistent with the fact that CpG methylation also varies in this taxon. Blattodea, Hemiptera and Hymenoptera, insects with the highest levels of CpG methylation^52^ had a marked bias for TGA, whereas such a bias was less pronounced in Coleoptera and Lepidoptera that have less significant CpG methylation. Significantly, species of the taxon Diptera, that have lost CpG methylation show practically no such bias (Table 2).

In summary, our studies have shown that methylation and hypermutability of CpG dinucleotides is the major source of the bias favoring TGA versus TAG as termination codons of protein-coding genes. The TGA versus TAG bias is generally valid across Eukarya that methylate CpG dinucleotides, however, in the case of Bacteria and Archaea changes in the function of stop codons have a greater influence on termination codon usage of proteins.

## Materials and methods

### Somatic mutation data

Cancer somatic mutation data were extracted from COSMIC v96 (COSMIC release v96, 31st May 2022), the Catalogue Of Somatic Mutations In Cancer (https://cancer.sanger.ac.uk/cosmic/download) which includes single nucleotide substitutions from targeted and genome wide screens, affecting the coding sequence of human genes.

Since we were interested in the selection forces that operate on the choice of stop codons during tumor evolution, only confirmed somatic, nonsense point mutations that arose during tumor evolution were included in our analyses. Accordingly, for all subsequent analyses we have retained only transcripts containing mutations that were annotated under’ Mutation description’ as Substitution—Nonsense and under’ Mutation somatic status’ as Confirmed Somatic, that is confirmed to be somatic in the experiment by sequencing both the tumor and a matched normal tissue from the same patient. As to’Sample Type, Tumor origin’: we have excluded mutation data from cell-lines, organoid-cultures, xenografts since they do not properly represent human tumor evolution at the organism level. To eliminate the influence of polymorphisms on the conclusions we retained only somatic mutations flagged ’n’ for SNPs. Finally, we have removed redundant data so that each unique nonsense substitution was represented only once in the dataset used in our analyses.

Although the COSMIC files provide information on the nature of the nucleotide substitution, its position in the coding sequence (under MUTATION_CDS e.g. „c.3342G > A”) and the effect of the substitution on the amino acid sequence of the protein (under MUTATION_AA, e.g. „p.R596*”), in most cases the identification of the mutant stop codons (TAA, TAG or TGA) requires the identification of the wild type codon and the position of the substitution within the codon, since in the case of several amino acids (e.g. Lys, Gln, Glu, Tyr, Ser, Leu, Trp) different substitutions may give rise to different nonsense codons (Supplementary table 1). For example, depending on the nucleotide position affected, a G > A substitution of Trp (TGG) converts it to TAG or TGA or, depending on the actual wild type codon used, an A > T substitution at the first position of the codon may convert a Lys (AAA, AAG) to TAA or TAG. To solve this problem, we have downloaded the files (All_COSMIC_Genes.fasta.gz) containing the nucleotide and amino acid sequences of the genes and—using the MUTATION_CDS information—have identified the sequences of the wild type sense codons and the mutant stop codons (see Supplementary file 1).

### Germline mutation data

Information on SNPs affecting the coding regions of human genes was obtained from the dbSNP database (https://www.ncbi.nlm.nih.gov/snp/). We have analyzed only variants that contained stop mutations that arose through single nucleotide substitution and have identified the sequences of the stop codons using the protocol described for the analysis of the somatic mutation data (Supplementary file 7).

### Substitution metrics

The 61 sense codons can undergo 549 single base substitutions and, depending on the wild type and mutant codon, substitutions can be assigned to the silent, missense or nonsense mutation category. Codons, however, differ significantly in the probability that their mutation would lead to nonsense mutation and whether the point mutation generates TAA, TAG or TGA. For example, single nucleotide substitutions of synonymous codons of Lys, Gln, Glu and Tyr can give rise to only TAA or TAG, whereas substitutions of codons of Arg, Gly and Cys can generate only stop codon TGA (Supplementary table 1). Since amino acids and synonymous codons do not occur with the same frequency in the coding region of human genes this may have a significant influence on the expected probability and choice of nonsense mutation. Furthermore, different classes of substitutions do not occur with equal probability; tumor tissues show a characteristic spectrum of substitutions classes^68,69^.

Substitutions are assigned to six classes (C > A, C > G, C > T, T > A, T > C, and T > G) referred to by the pyrimidine of the mutated Watson–Crick base pair. It is of crucial importance to take differences in the probability of the six mutation classes into account since—due to the unique structure of the genetic code—the six types of substitutions differ markedly in the probability that they would lead to TAA, TAG or TGA nonsense mutation. To take into account the influence of codon frequencies and mutation bias on the frequency of the three nonsense mutations of human proteins in the absence of selection, we have followed the procedure described previously^22^.

For these calculations, we have downloaded the coding sequences of human protein coding genes (All_COSMIC_Genes.fasta.gz) from the COSMIC database (https://cancer.sanger.ac.uk/cosmic) and their codon usage and amino acid composition were determined using the SMS server (https://www.bioinformatics.org/sms2/codon_usage.html)^70^.

To correct for differences in probability of different substitutions classes, we have calculated the contribution of the C > A, C > G, C > T, T > A, T > C, and T > G mutations to the pattern of single base substitutions in tumors using the files ‘Mutational Signatures v3.1’ and ‘Attributions of the SBS Signatures to Mutations in Tumors’ downloaded from the COSMIC website (https://cancer.sanger.ac.uk/ cosmic/signatures/SBS/index.tt). The expected fTAA_exp_, fTAG_exp_, and fTGA_exp_ values were calculated using the average values of the six substitution types observed across tumors. In the case of germline cells, we have also calculated the expected fTAA_exp_, fTAG_exp_, and fTGA_exp_ values using the mutation probabilities characteristic of these cells. It has been shown earlier that the human germline mutation spectrum can be recapitulated by a combination of the cancer signatures SBS1 and SBS5^71–73^. In the present work, we have combined the effect of mutation signatures SBS1 and SBS5 on the germline mutation spectrum of proteins according to the formula (0.1 SBS1 + 0.9 SBS5) recommended by Heredia-Genestar et al.^73^.

### Detection of selection signals in tumor tissues

 For each gene we have determined the fractions of the three types of nonsense mutations observed in tumor tissues (fTAA_obs_, fTAG_obs_ and fTGA_obs_) as well as those expected in the absence of selection (fTAA_exp_, fTAG_exp_, and fTGA_exp_). The data for the four different gene groups, tumor suppressor genes (TSGs), oncogenes (OGs), tumor essential genes (TEGs) and passenger genes (PGs) were analyzed separately as they are known to differ in their sensitivity to nonsense substitutions^22^.

### Lists of genes analyzed

We have analyzed four different groups of human protein-coding genes, known to differ in selection for or against nonsense mutations in cancer. As the gold standard of ’known’ cancer genes we have used the lists of oncogenes (OGs) and tumor suppressor genes (TSGs) identified by Vogelstein et al.^23^. The list of tumor essential genes (TEGs) consisted of genes identified by Bányai et al.^22^. As a control group, we have used the list of passenger genes (PGs) characterized by Bányai et al.^24^. The same sets of genes were also subjected to an analysis of germline substitutions resulting in nonsense mutations.

The lists of de novo genes analysed in the present work to calculate their pattern of termination codons included murine genes^36^, human genes^37–39^, as well as genes from *S. cerevisiae*^40^, *D. melanogaster*^41–43^ and *C. elegans*^44,45^.

### Analysis of nonsense codon usage of proteins

We have used the Codon Usage Database (https://www.kazusa.or.jp/codon/) ^74^ as a source to calculate the pattern of termination codons of the coding genomes of organisms. The patterns of hidden, out of frame stop codons were determined with the Codon Usage tool of the Sequence Manipulation Suite^70^ The termination codon spectra of species representing Archaea, Bacteria and Eukarya were obtained from the Codon Statistics Database (http://codonstatsdb.unr.edu/) ^57^.

### Statistical analyses

The statistical package of Origin 2018 was used for all data processing and statistical analysis. We report details of statistical tests in the Supplementary files of the respective sections. Statistical significance was set as a p value of < 0.05.

### Supplementary Information

Supplementary Information 1.

Supplementary Information 2.

Supplementary Information 3.

Supplementary Information 4.

Supplementary Information 5.

Supplementary Information 6.

Supplementary Information 7.

Supplementary Information 8.

Supplementary Information 9.

Supplementary Information 10.

Supplementary Information 11.

## Data Availability

All data generated or analysed during this study are included in this published article [and its supplementary information files].

## References

[1] Trotta E (2016). Selective forces and mutational biases drive stop codon usage in the human genome: A comparison with sense codon usage. BMC Genom..

[2] Povolotskaya IS, Kondrashov FA, Ledda A, Vlasov PK (2012). Stop codons in bacteria are not selectively equivalent. Biol. Direct..

[3] Korkmaz G, Holm M, Wiens T, Sanyal S (2014). Comprehensive analysis of stop codon usage in bacteria and its correlation with release factor abundance. J. Biol. Chem..

[4] Ho AT, Hurst LD (2022). Stop codon usage as a window into genome evolution: Mutation, selection, biased gene conversion and the TAG paradox. Genome Biol. Evol..

[5] Belin D, Puigbò P (2022). Why is the UAG (Amber) stop codon almost absent in highly expressed bacterial genes?. Life (Basel)..

[6] Belinky F, Babenko VN, Rogozin IB, Koonin EV (2018). Purifying and positive selection in the evolution of stop codons. Sci. Rep..

[7] Stiebler AC (2014). Ribosomal readthrough at a short UGA stop codon context triggers dual localization of metabolic enzymes in Fungi and animals. PLoS Genet..

[8] Anzalone AV, Zairis S, Lin AJ, Rabadan R, Cornish VW (2019). Interrogation of eukaryotic stop codon readthrough signals by in vitro RNA selection. Biochemistry.

[9] Schilff M, Sargsyan Y, Hofhuis J, Thoms S (2021). Stop codon context-specific induction of translational readthrough. Biomolecules.

[10] Manjunath LE, Singh A, Som S, Eswarappa SM (2022). Mammalian proteome expansion by stop codon readthrough. Wiley Interdiscip. Rev. RNA.

[11] Jungreis I (2011). Evidence of abundant stop codon readthrough in Drosophila and other metazoa. Genome Res..

[12] Jungreis I (2016). Evolutionary dynamics of abundant stop codon readthrough. Mol. Biol. Evol..

[13] Schueren F, Thoms S (2016). Functional translational readthrough: A systems biology perspective. PLoS Genet..

[14] Belinky F, Ganguly I, Poliakov E, Yurchenko V, Rogozin IB (2021). Analysis of stop codons within prokaryotic protein-coding genes suggests frequent readthrough events. Int. J. Mol. Sci..

[15] Fan Y (2017). Heterogeneity of stop codon readthrough in single bacterial cells and implications for population fitness. Mol. Cell.

[16] Zhang H (2020). Metabolic stress promotes stop-codon readthrough and phenotypic heterogeneity. Proc. Natl. Acad. Sci. USA.

[17] Hofhuis J (2016). The functional readthrough extension of malate dehydrogenase reveals a modification of the genetic code. Open Biol..

[18] Schueren F (2014). Peroxisomal lactate dehydrogenase is generated by translational readthrough in mammals. Elife.

[19] Li C, Zhang J (2019). Stop-codon read-through arises largely from molecular errors and is generally nonadaptive. PLoS Genet..

[20] Arribere JA (2016). Translation readthrough mitigation. Nature.

[21] Shibata N (2015). Degradation of stop codon read-through mutant proteins via the ubiquitin-proteasome system causes hereditary disorders. J Biol Chem..

[22] Bányai L, Trexler M, Kerekes K, Csuka O, Patthy L (2021). Use of signals of positive and negative selection to distinguish cancer genes and passenger genes. Elife.

[23] Vogelstein B (2013). Cancer genome landscapes. Science.

[24] Bányai L, Trexler M, Patthy L (2022). Use of publication dynamics to distinguish cancer genes and bystander genes. Genes (Basel)..

[25] Krawczak M, Ball EV, Cooper DN (1998). Neighboring-nucleotide effects on the rates of germ-line single-base-pair substitution in human genes. Am. J. Hum. Genet..

[26] Carvunis AR (2012). Proto-genes and de novo gene birth. Nature.

[27] Bornberg-Bauer E, Hlouchova K, Lange A (2021). Structure and function of naturally evolved de novo proteins. Curr. Opin. Struct. Biol..

[28] Neme R, Tautz D (2013). Phylogenetic patterns of emergence of new genes support a model of frequent de novo evolution. BMC Genom..

[29] McLysaght A, Hurst LD (2016). Open questions in the study of de novo genes: What, how and why. Nat. Rev. Genet..

[30] Karlin S, Ladunga I, Blaisdell BE (1994). Heterogeneity of genomes: measures and values. Proc. Natl. Acad. Sci. USA.

[31] Simmen MW (2008). Genome-scale relationships between cytosine methylation and dinucleotide abundances in animals. Genomics.

[32] Simmonds P, Xia W, Baillie JK, McKinnon K (2013). Modelling mutational and selection pressures on dinucleotides in eukaryotic phyla—selection against CpG and UpA in cytoplasmically expressed RNA and in RNA viruses. BMC Genom..

[33] Misawa K, Kamatani N, Kikuno RF (2008). The universal trend of amino acid gain-loss is caused by CpG hypermutability. J. Mol. Evol..

[34] Misawa K, Kikuno RF (2009). Evaluation of the effect of CpG hypermutability on human codon substitution. Gene.

[35] Dixon GB, Bay LK, Matz MV (2016). Evolutionary consequences of DNA methylation in a basal metazoan. Mol. Biol. Evol..

[36] Ruiz-Orera J, Verdaguer-Grau P, Villanueva-Cañas JL, Messeguer X, Albà MM (2018). Translation of neutrally evolving peptides provides a basis for de novo gene evolution. Nat. Ecol. Evol..

[37] Xie C (2012). Hominoid-specific de novo protein-coding genes originating from long non-coding RNAs. PLoS Genet..

[38] An NA (2023). De novo genes with an lncRNA origin encode unique human brain developmental functionality. Nat. Ecol. Evol..

[39] Sandmann CL (2023). Evolutionary origins and interactomes of human, young microproteins and small peptides translated from short open reading frames. Mol Cell..

[40] Blevins WR (2021). Uncovering de novo gene birth in yeast using deep transcriptomics. Nat. Commun..

[41] Lee BY, Kim J, Lee J (2022). Intraspecific de novo gene birth revealed by presence-absence variant genes in Caenorhabditis elegans. NAR Genom Bioinform..

[42] Zhang W, Gao Y, Long M, Shen B (2019). Origination and evolution of orphan genes and de novo genes in the genome of Caenorhabditis elegans. Sci. China Life Sci..

[43] Reinhardt JA (2013). De novo ORFs in Drosophila are important to organismal fitness and evolved rapidly from previously non-coding sequences. PLoS Genet..

[44] Heames B, Schmitz J, Bornberg-Bauer E (2020). A continuum of evolving de novo genes drives protein-coding novelty in drosophila. J Mol Evol..

[45] Begun DJ, Lindfors HA, Thompson ME, Holloway AK (2006). Recently evolved genes identified from Drosophila yakuba and D. erecta accessory gland expressed sequence tags. Genetics.

[46] Wojciechowski M, Czapinska H, Bochtler M (2013). CpG underrepresentation and the bacterial CpG-specific DNA methyltransferase M.MpeI. Proc. Natl. Acad. Sci. USA.

[47] Wong TY (2008). Role of premature stop codons in bacterial evolution. J. Bacteriol..

[48] Morgens DW, Chang CH, Cavalcanti AR (2013). Ambushing the Ambush Hypothesis: Predicting and evaluating off-frame codon frequencies in prokaryotic genomes. BMC Genom..

[49] Jeltsch A (2010). Molecular biology. Phylogeny of methylomes. Science.

[50] Schmitz RJ, Lewis ZA, Goll MG (2019). DNA methylation: Shared and divergent features across eukaryotes. Trends Genet..

[51] Engelhardt J, Scheer O, Stadler PF, Prohaska SJ (2022). Evolution of DNA methylation across ecdysozoa. J. Mol. Evol..

[52] Bewick AJ, Vogel KJ, Moore AJ, Schmitz RJ (2017). Evolution of DNA methylation across insects. Mol Biol Evol..

[53] Gao F (2012). Differential DNA methylation in discrete developmental stages of the parasitic nematode Trichinella spiralis. Genome Biol..

[54] Simpson VJ, Johnson TE, Hammen RF (1986). Caenorhabditis elegans DNA does not contain 5-methylcytosine at any time during development or aging. Nucl. Acids Res..

[55] Proffitt JH, Davie JR, Swinton D, Hattman S (1984). 5-Methylcytosine is not detectable in Saccharomyces cerevisiae DNA. Mol. Cell Biol..

[56] Ying H, Huttley G (2011). Exploiting CpG hypermutability to identify phenotypically significant variation within human protein-coding genes. Genome Biol. Evol..

[57] Subramanian K, Payne B, Feyertag F, Alvarez-Ponce D (2022). The codon statistics database: A database of codon usage bias. Mol. Biol. Evol..

[58] Patil NA, Basu B, Deobagkar DD, Apte SK, Deobagkar DN (2017). Putative DNA modification methylase DR_C0020 of Deinococcus radiodurans is an atypical SAM dependent C-5 cytosine DNA methylase. Biochim Biophys Acta Gen Subj..

[59] Yamao F (1985). UGA is read as tryptophan in Mycoplasma capricolum. Proc. Natl. Acad. Sci. USA.

[60] Citti C, Maréchal-Drouard L, Saillar C, Weil JH, Bové JM (1992). Spiroplasma citri UGG and UGA tryptophan codons: sequence of the two tryptophanyl-tRNAs and organization of the corresponding genes. J. Bacteriol..

[61] Campbell JH (2013). UGA is an additional glycine codon in uncultured SR1 bacteria from the human microbiota. Proc. Natl. Acad. Sci. USA.

[62] Hanke A (2014). Recoding of the stop codon UGA to glycine by a BD1-5/SN-2 bacterium and niche partitioning between Alpha- and Gammaproteobacteria in a tidal sediment microbial community naturally selected in a laboratory chemostat. Front. Microbiol..

[63] Borrel G (2014). Unique characteristics of the pyrrolysine system in the 7th order of methanogens: Implications for the evolution of a genetic code expansion cassette. Archaea.

[64] Guo LT (2022). Ancestral archaea expanded the genetic code with pyrrolysine. J. Biol. Chem..

[65] Shimizu TS, Takahashi K, Tomita M (1997). CpG distribution patterns in methylated and non-methylated species. Gene.

[66] Albalat R, Martí-Solans J, Cañestro C (2012). DNA methylation in amphioxus: From ancestral functions to new roles in vertebrates. Brief Funct. Genom..

[67] Keller TE, Han P, Yi SV (2016). Evolutionary Transition of Promoter and Gene Body DNA Methylation across Invertebrate-Vertebrate Boundary. Mol. Biol. Evol..

[68] Alexandrov LB (2013). Signatures of mutational processes in human cancer. Nature.

[69] Alexandrov LB (2020). The repertoire of mutational signatures in human cancer. Nature.

[70] Stothard P (2000). The sequence manipulation suite: JavaScript programs for analyzing and formatting protein and DNA sequences. Biotechniques.

[71] Alexandrov LB (2015). Clock-like mutational processes in human somatic cells. Nat Genet..

[72] Rahbari R (2016). Timing, rates and spectra of human germline mutation. Nat Genet..

[73] Heredia-Genestar JM, Marquès-Bonet T, Juan D, Navarro A (2020). Extreme differences between human germline and tumor mutation densities are driven by ancestral human-specific deviations. Nat. Commun..

[74] Nakamura Y, Gojobori T, Ikemura T (2000). Codon usage tabulated from international DNA sequence databases: status for the year 2000. Nucl. Acids Res..

